# Impact of selenium biofortification on production characteristics of forages grown following standard management practices in Oregon

**DOI:** 10.3389/fpls.2023.1121605

**Published:** 2023-03-31

**Authors:** Jean A. Hall, Gerd Bobe, Shelby J. Filley, Mylen G. Bohle, Gene J. Pirelli, Guogie Wang, T. Zane Davis, Gary S. Bañuelos

**Affiliations:** ^1^ Department of Biomedical Sciences, Carlson College of Veterinary Medicine, Oregon State University, Corvallis, OR, United States; ^2^ Department of Animal and Rangeland Sciences, College of Agricultural Sciences, Oregon State University, Corvallis, OR, United States; ^3^ Linus Pauling Institute, Oregon State University, Corvallis, OR, United States; ^4^ Department of Crop and Soil Science, College of Agricultural Sciences, Oregon State University, Corvallis, OR, United States; ^5^ United States Department of Agriculture (USDA), Agricultural Research Service-Poisonous Plant Research Lab, Logan, UT, United States; ^6^ United States Department of Agriculture (USDA), Agricultural Research Service-San Joaquin Valley Agricultural Sciences Center, Parlier, CA, United States

**Keywords:** forage fertilization, grasses, legumes, nitrogen, selenium yield, selenium agronomic efficiency, sulfur

## Abstract

**Introduction:**

Low selenium (Se) concentrations in soils and plants pose a health risk for ruminants consuming locally-grown forages. Previous studies have shown that Se concentrations in forages can be increased using soil-applied selenate amendments. However, the effects of foliar selenate amendments applied with traditional nitrogen-phosphorus-potassium-sulfur (NPKS) fertilizers on forage yields, and nutrient contents, and agronomic efficiencies are unknown.

**Methods:**

Using a split plot design, we determined the effects of springtime sodium selenate foliar amendment rates (0, 45, and 90 g Se ha^-1^) and NPKS application (none, NPK for grasses/PK for alfalfa, and NPKS/PKS fertilization at amounts adapted to meet local forage and soil requirements) on forage growth and N, S, and Se concentrations, yields, and agronomic efficiencies. This 2-year study was conducted across Oregon on four representative forage fields: orchardgrass (*Dactylis glomerata* L.) in Terrebonne (central Oregon), grass-clover mixture in Roseburg (southwestern Oregon), and both grass mixture and alfalfa (*Medicago sativa* L.) fields in Union (eastern Oregon).

**Results:**

Grasses grew poorly and were low in N content without NPK fertilization. Fertilization with NPK/PK promoted forage growth, increased forage N concentrations, and had to be co-applied with S when plant available S was low. Without Se amendment, forage Se concentrations were low and further decreased with NPKS/PKS fertilization. Selenate amendment linearly increased forage Se concentration without adversely affecting forage yields, N and S concentrations, or N and S agronomic efficiencies.

**Discussion:**

Importantly, S fertilization did not interfere with Se uptake in Se amended plots. In conclusion, co-application of NPKS/PKS fertilizers and foliar sodium selenate in springtime is an effective strategy to increase forage total Se concentrations, while maintaining optimal growth and quality of Oregon forages.

## 1 Introduction

Forage serves as an inexpensive, primary feed source for ruminant livestock operations (Schroeder, 2018). A challenge for livestock in many parts of the world, including Oregon, is that plant-available selenium (Se) concentrations are low (<0.1 mg Se/kg DM) in soils and locally produced forages (Oldfield, 2001). Selenium is an essential micronutrient for animals. Livestock consuming locally produced forages are susceptible to Se deficiency resulting in poor health and growth unless Se supplementation is provided. The health of livestock can be improved by feeding Se biofortified forages, a practice known as Se agronomic biofortification (Schiavon et al., 2020). Selenate amendment of pastures increases Se concentrations of forages, which, in turn, improves Se concentrations in forage-consuming cattle and sheep (Hall et al., 2009; Hall et al., 2011a).

A comparison across a limited number of studies varying the amount of Se applied per hectare suggests that there is linear increase in Se content of forage in response to Se dosage (Hall et al., 2009; Hall et al., 2013a; Wallace et al., 2017). The currently recommended Se application rates are 12.4-24.7 g Se ha^-1^, as reviewed in (Brummer et al., 2014). However, we have previously shown health benefits in cattle and sheep fed supranutritional Se concentrations from forages grown on low-Se soil amended with 22.5, 45, and 90 g Se ha^-1^. Thus, we are interested in Se dosages at concentrations that are higher than currently recommended by the US-FDA for preventing clinical Se deficiency (i.e., supranutritional dosages). At these higher concentrations we have observed improved production and fewer diseases without adverse health outcomes. We have evaluated supranutritional Se supplementation in Se-replete cattle and sheep throughout production stages, as well as during specific high-demand stages (e.g., during the backgrounding period before transport to the feedlot, and during the last 2–3 months of gestation) and have observed production and immune function improvements with both strategies in animals at the highest supplementation levels (Hall et al., 2011b; Stewart et al., 2012; Hall et al., 2013a; Hall et al., 2013b; Hugejiletu et al., 2013; Hall et al., 2014a; Hall et al., 2014b; Hall et al., 2017; Hall et al., 2020).

In contrast to animals, Se is not an essential nutrient for plants because plants utilize sulfur (S) rather than Se for their redox chemistry (reviewed in (White, 2018)). Plants do not have a SeCys-specific codon for peptide and protein synthesis, but rather both S and Se can incorporate interchangeably into the N-containing amino acids cysteine/SeCys and methionine/SeMet. In micro-mineral amounts (<100 mg Se kg^-1^ plant DM), Se may benefit plant health and growth because SeCys can more quickly and easily reverse protein oxidation and control redox processes than cysteine (Boldrin et al., 2016; White, 2018) thereby improving forage quality. In macro-mineral amounts (>100 mg kg^-1^ plant DM), Se is toxic for non-seleniferous plants, such as forage legumes and grasses (White, 2016).

Optimal forage production also depends on applying NPKS fertilizers (Moore et al., 2019). Fertilization with the macronutrients nitrogen (N) and S, or both, is an important part of forage nutrient management (Moore et al., 2019). Insufficient N results in low forage yield and quality, indicated by pale green colored leaves and low forage grass DM N concentrations (<2-2.5% plant DM) (Moore et al., 2019). Forage grasses depend on N fertilization, using ammonium and nitrate compounds for plant growth, protein synthesis, and tillering (Moore et al., 2019). In contrast, bacteria in the root region of legumes convert N gas from the air to ammonium-N compounds for plant growth and protein synthesis (Russelle et al., 1994). Nonetheless, grasses outcompete legumes in growth when fields are fertilized with N in the form of nitrate or ammonium-N compounds (Moore et al., 2019). To optimize forage yield and quality, N fertilization of grasses often requires co-application of S to satisfy the grasses’ need for the S-containing amino acids cysteine and methionine for protein synthesis (Lancaster et al., 1971; Bolton et al., 1976; Aulakh, 2003; Moore et al., 2019). Sulfur fertilization using sulfate components provides plants with sulfate for root absorption. Forage S concentrations < 0.2% or a N:S ratio > 10 may indicate S deficiency (Moore et al., 2019; White et al., 2021).

Less research has focused on whether concurrent use of NPKS fertilizers affect Se biofortification (Li et al., 2007; Duncan et al., 2017). There are concerns that S fertilization may exacerbate forage Se deficiencies, because selenate and sulfate compete for the same root membrane transporter, the expression of which is increased during S depletion (Pratley and McFarlane, 1974; Lauchli, 1993; Gupta and Macleod, 1994; White et al., 2004; Li et al., 2008; Luo et al., 2019; Deng et al., 2021). However, foliar selenate amendment may prevent the competition of Se and sulfur for root absorption (Ros et al., 2016; Ramkissoon et al., 2019; Schiavon et al., 2022).

Our objective is to make application of foliar selenate amendment a standard nutrient management practice for Oregon’s Se-deficient pastures. To achieve our objective, we investigated over two years the effects of springtime sodium selenate foliar application (0, 45, and 90 g Se ha^-1^) and nitrogen, phosphorus, potassium (NPK) fertilization, alone or in combination with S (NPKS), on forages grown throughout Oregon. We previously reported on the effect of selenate amendment and NPKS fertilization on forage Se concentrations, as well as Se species composition (Wang et al., 2021). In the current study, we focus on the effects of selenate amendment and NPKS fertilization on total forage biomass (indicative of forage growth), nutrient concentrations (indicative of forage and feed quality), nutrient yields (indicative of nutrient uptake), and nutrient agronomic efficiencies (indicative of Se amendment or fertilizer N and S use). We hypothesized, given the much lower amounts of selenate applied (*vs*. N, P, K and S), that application of standard NPKS fertilizers to Oregon’s Se-deficient pastures should not interfere with Se yields or Se agronomic efficiencies.

## 2 Materials and methods

### 2.1 Experimental design and agronomic field management

**Figure 1:**
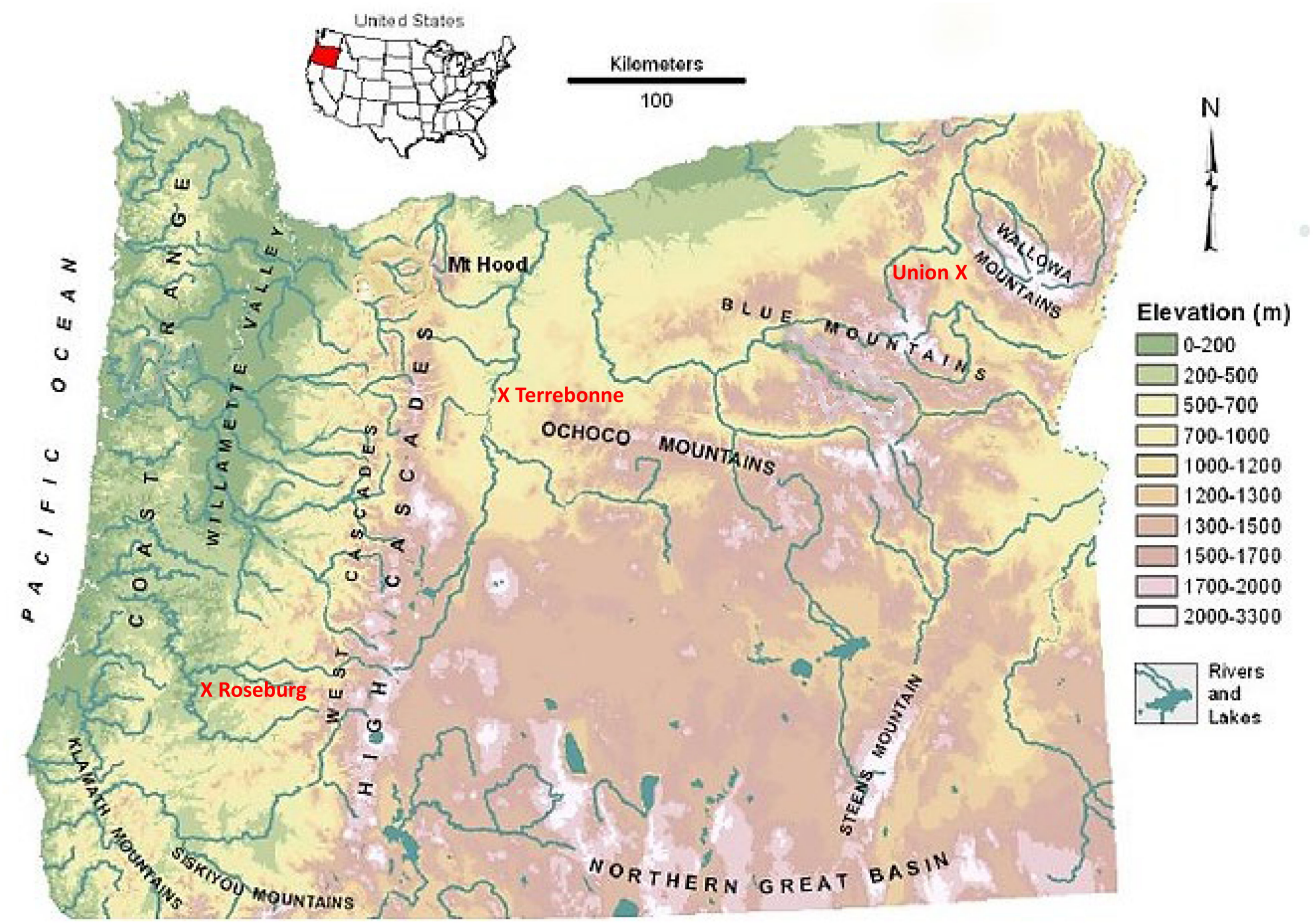
Map showing locations of forage sites at Union (eastern Oregon), Terrebonne (central Oregon), and Roseburg (western Oregon). Adapted from Leibowitz et al. (2014).

The study was conducted from 2017 to 2019 using four irrigated hayfields at three different climatic sites, Roseburg (43.2°N, 123.3°W, and 161 m asl.) in southwestern Oregon, Terrebonne (44.4°N, 121.2°W, and 851 m asl.) in central Oregon, and Union (45.2°N, 117.9°W, and 853 m asl.) in eastern Oregon (**Figure 1**). The average growing seasons are much shorter in central (80 days) and eastern (119 days) Oregon compared with southwestern (182 days) Oregon. We collected rainfall and temperature data during the study period (May 2017 to May 2019): average rainfall and temperatures were higher in southwestern Oregon (Roseburg: 77 mm mo^-1^ and 11.4°C) compared with central and eastern Oregon (Terrebonne: 20 mm mo^-1^ and 8.3°C; Union: 40 mm mo^-1^ and 8.1°C). Whereas maximal monthly temperatures in summer were similar across the three sites (Roseburg: 34°C; Terrebonne: 32°C; Union: 33°C), average monthly temperatures in winter were milder in southwestern Oregon (Roseburg: 3°C) compared with central and eastern Oregon (-2°C). Rainfall was seasonal with nearly no precipitation (<1 mm mo^-1^) in July and August, requiring irrigation during the summer to provide plants with sufficient water to off-set evapotranspiration. In central and southwestern Oregon, the growing season rainfall was on average in 2017, but unusually dry in 2018 (Terrebonne: 124 mm in 2017 and 15 mm in 2018; Roseburg: 60 mm in 2017 and 26 mm in 2018), with no differences in eastern Oregon (Union: 92 mm in 2017 and 91 mm in 2018).

The soil types were loam in Roseburg (11.0% clay, 62.8% sand, and 26.2% silt), sandy loam in Terrebonne (9.4% clay, 59.8% sand, and 30.8% silt), and silt loam in Union (22.5% clay, 9.5% sand, and 68.0% silt) (**Table 1**). The soil organic matter content was low in central and southwestern Oregon (Roseburg: 1%; Terrebonne: 1.7%) and high in Union (4.7-6.6% in the alfalfa field and 4.9-5.5% in the grass field). The cation exchange capacity (CEC) was higher in Union (alfalfa field: 36.2-36.7 mEq 100 g^-1^; grass field: 38.0-41.1 mEq 100 g^-1^) compared with Terrebonne (13.8-17.0 mEq 100 g^-1^) and Roseburg (18.0 mEq 100 g^-1^). The soil pH values were acidic in Roseburg (pH: 5.6) and Terrebonne (pH: 5.5-5.7) and close to neutral in Union (grass field pH: 6.5-6.6; alfalfa field pH: 7.0-7.2). The soluble salt (SS) content of the soil was low at all three sites: Roseburg (0.1 mmhos cm^-1^), Terrebonne (0.1-0.3 mmhos cm^-1^), and Union (both 0.3 mmhos cm^-1^). The total soil Se concentration was also low at all three sites: Roseburg (0.18 mg Se kg^-1^ DM), Terrebonne (0.12 mg Se kg^-1^ DM), and Union (0.15 mg Se kg^-1^ DM).

**Table 1:** Soil analysis for forage sites at Union (eastern Oregon), Terrebonne (central Oregon), and Roseburg (western Oregon)^1^.

Site/Forage/Date	Plot	pH	pH Buffered	OM^2^	SS	CEC	P	K	Mg	Ca	Na	NO_3_	NH_4_	S
				g 100g^-1^	dS m^-1^	Cmol(+) kg^-1^	mg kg^-1^	mg kg^-1^	mg kg^-1^	mg kg^-1^	mg kg^-1^	mg kg^-1^	mg kg^-1^	mg kg^-1^
Union/Alfalfa	Soil type: silt loam (22.5% clay, 9.5% sand, and 68.0% silt)						Olsen^3^							
Guidelines^4,5,6^		6-8.2	NA^1^	NA	<1.0	NA	20	200	NA	NA	<10.0	NA	NA	15
19.04.2017	Baseline	7.0	7.0	4.2	0.2	31.3	23	178	4	11.5	0.3	14.4	18.25	11
16.04.2018	None	7.0	6.9	4.7	0.3	36.7	26	208	4.55	13.4	0.36	22	19	21.6
16.04.2018	PK	7.1	7.1	6.6	0.3	36.2	25	191	4.55	13.15	0.33	28.5	22.5	23.2
16.04.2018	PKS	7.2	7.1	4.7	0.3	36.3	21	156	4.65	13.1	0.35	22	21	12.9
Union/Grasses	Soil type: silt loam (22.5% clay, 9.5% sand, and 68.0% silt)						Olsen^3^							
Guidelines^4,7^		5.6	NA	NA	<1.0	NA	10	150	NA	NA	<10.0	7	NA	NA
19.04.2017	Baseline	6.3	6.6	5.6	0.2	40.3	32	205	5	12	0.3	18.5	22	16
16.04.2018	None	6.6	6.6	4.9	0.3	38.0	29	231	5.45	13.05	0.33	11.5	18.5	16.7
16.04.2018	NPK	6.6	6.7	4.9	0.3	38.6	22	207	5.7	13.2	0.32	8.5	16.5	15.2
16.04.2018	NPKS	6.5	6.8	5.5	0.3	41.1	29	193	5.5	13.15	0.31	10.5	27	14.7
Terrebonne Orchard Grass	Soil type: sandy loam (9.4% clay, 59.8% sand, and 30.8% silt)						Olsen^3^							
Guidelines^4,7^		5.6	NA	NA	<1.0	NA	10	150	NA	NA	<10.0	7	NA	NA
6.04.2017	Baseline	5.6	6.7	1.7	0.1	13.8	23	116	1.5	3.1	0.26	2.5	4	9.7
24.04.2018	None	5.7	6.6	ND	0.3	17.0	27	147	1.85	3.75	0.38	14.5	25.5	14.5
24.04.2018	NPK	5.6	6.6	ND	0.3	16.4	27	123	1.75	3.6	0.35	9	17	11.9
24.04.2018	NPKS	5.6	6.9	ND	0.3	13.9	29	120	1.8	3.6	0.36	10	21.5	13.6
Roseburg Grass-Clover	Soil type: loam (11.0% clay, 62.8% sand, and 26.2% silt)						Bray^3^							
Guidelines^4,8^		5.5	NA	>2.0	<1.0	NA	30	200	0.8	5.0	<10.0	NA	NA	NA
12.05.2017	Baseline	5.6	6.8	ND	0.1	18.0	43	186	2.2	4.95	0.13	ND	ND	ND
02.04.2018	None	6.1	6.5	ND	0.2	20.2	9	159	2.15	4.65	0.17	ND	ND	ND
02.04.2018	NPK	6.1	6.7	ND	0.1	18.4	8	161	2.2	4.7	0.16	ND	ND	ND
02.04.2018	NPKS	6.0	6.8	ND	0.2	17.4	8	138	2.2	4.75	0.17	ND	ND	ND

^1^Soil samples were taken 0 to 15.24 cm in Roseburg and 0 to 30.48 cm in Union and Terrebonne. Samples were analyzed by AgSource Laboratories in Umatilla (Oregon).

^2^OM, organic matter; SS, soluble salts (measure of the amount of nutrients in solution in the form of electric conductivity dS/m); CEC, cation exchange capacity (measure of the amount of cations a soil can adsorb by cation exchange, usually expressed as cmol(+) kg^-1^); P, phosphorus; K, potassium; Mg, magnesium; Ca, calcium; Na, sodium; NO_3_, nitrate; NH_4_, ammonium; S, sulfate-sulfur; NA, not available; ND, not determined.

^3^Olsen and Bray indicate methods for phosphorus determination. The Bray test is unreliable at soil pH > 7.45. Olsen value = 3.5 + (0.42 x Bray value). The Olsen sodium bicarbonate extraction method is used for soils east of the Cascade Mountain Range (Union and Terrebonne) and the Bray P1 extraction method is used for soils west of the Cascade Mountain Range (Roseburg, OR). Soil, Plant and Water Reference Methods for the Western Region (Miller et al., 2013).

^4^Soil Test Interpretation Guide (Horneck et al., 2011).

^5^Nutrient Management Guide for Dryland and Irrigated Alfalfa in the Inland Northwest (Koenig et al., 2009).

^6^Alfalfa (eastern Oregon – east of the Cascades) (Gardner et al., 1981).

^7^Irrigated clover-grass pastures: eastern Oregon – east of Cascades (Gardner et al., 2000).

^8^Nutrient Management Guide for Western Oregon and Washington Pastures (Moore et al., 2019).

The diverse climatic and edaphic conditions impact the forage types that can be profitably grown at each site. The four forage fields were alfalfa (*Medicago sativa* L.) in Union; orchardgrass (*Dactylis glomeruata* L.) in Terrebonne; a grass mixture [primarily tall fescue (*Lolium arundinaceum* (Schreb.) Darbysh. formerly *Festuca arundinacea* (Schreb.) and orchardgrass] in Union; and a 50:50 grass-clover mixture [dominated with tall fescue, orchardgrass, perennial ryegrass (*Lolium perenne* L.), white clover (*Trifolium repens* L.), and subterranean clover (*Trifolium subterraneum* L.)] in Roseburg.

**Figure 2:**
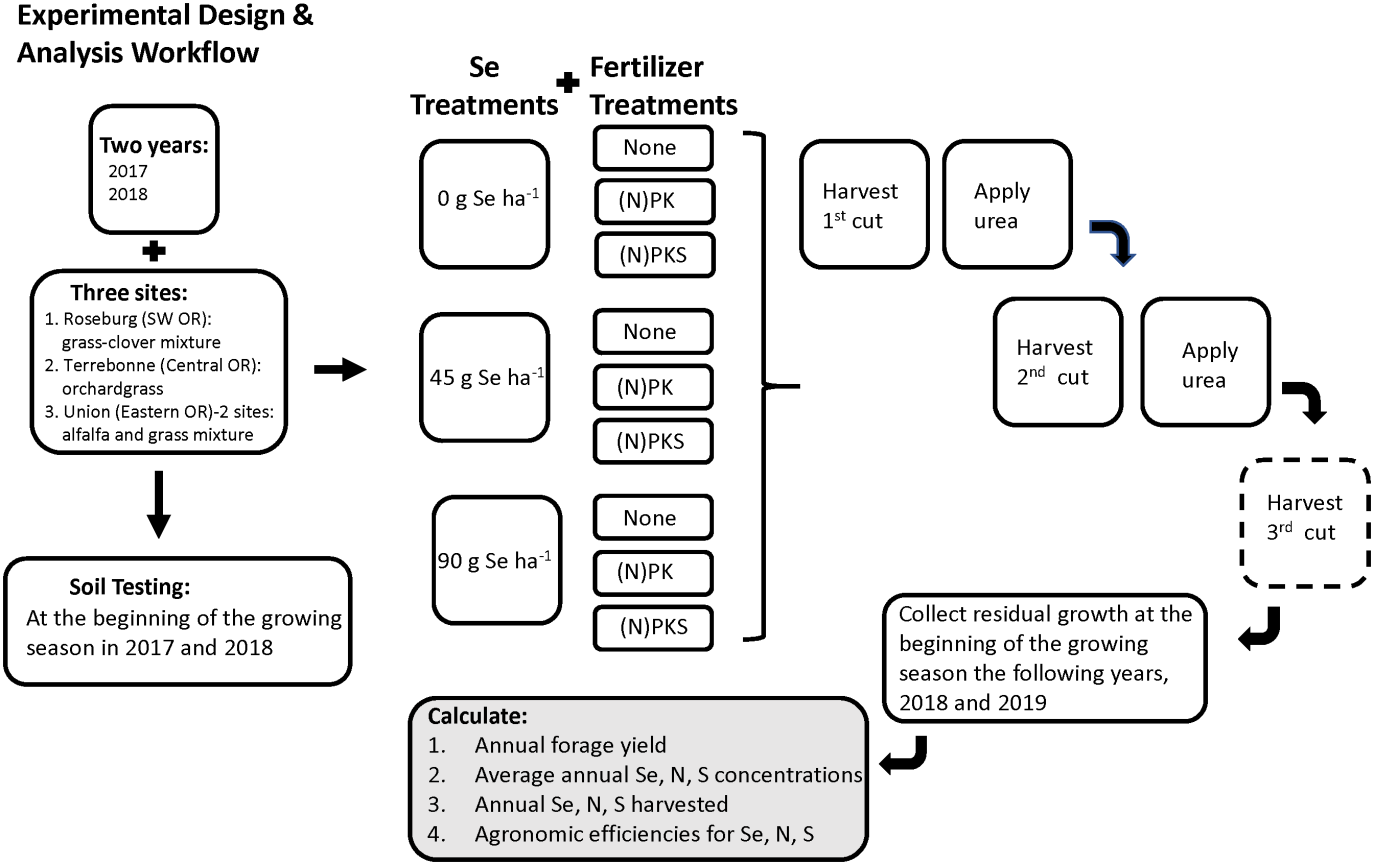
Experimental design and analysis workflow for soil sample collection, Se and fertilizer treatments, and forage sample cuttings taken at each of the testing sites in Oregon: Union (eastern Oregon), Terrebonne (central Oregon), and Roseburg (western Oregon). Each Se by fertilizer treatment combination was performed in triplicate at each forage species site. The third cut harvest was collected when possible.

At each location, the experiment was laid out as a split-plot design with three replications, with selenate application rate as whole plot and NPKS fertilization protocol as split plot (**Figure 2**). The plot dimensions were 6.0 m × 1.5 m in Roseburg, 4.5 m × 1.5 m in Terrebonne, and 6.1 m × 2.4 m in Union, which was based on field location and research machinery availability. Sodium selenate (RETORTE Ulrich Scharrer GmbH, Röthenbach, Germany) was applied at rates of 0, 45, or 90 g Se ha^-1^ to the same field whole plots on May 5, 2017 and May 16, 2018 in Roseburg; April 21, 2017 and May 4, 2018 in Terrebonne; and May 2, 2017 and 2018 in Union (**Table 2**). Plants were 5 to 10 cm high and in the tillering phase of the vegetative stage with leaves covering most of the soil. First, sodium selenate was dissolved in water (500 mL of water per plot). To uniformly cover the entire treatment area, we calibrated prior to application a back-pack sprayer with a time/speed calibration method. Sufficient water was added so that the treatment could be applied in a consistent manner using a spray pressure and walking speed that was easily maintained by the applicator. The aqueous sodium selenate solution was applied with a backpack sprayer fitted with a precision nozzle to deliver the specified application rate.

**Table 2:** Timeline for soil sample collection, Se and fertilizer treatments, and forage sample cuttings taken at each of the testing sites in Oregon: Union (eastern Oregon), Terrebonne (central Oregon), and Roseburg (western Oregon).

	Roseburg	Terrebonne	Union	Union
	Grass-clover mixture	Orchard grass	Alfalfa	Grass mixture
Baseline soil samples collected	May 4, 2017	April 6, 2017	April 15, 2017	April 15, 2017
Fertilizer treatments, 2017	May 4, 2017	April 21, 2017	April 29, 2017	April 29, 2017
Se treatments, 2017	May 5, 2017	April 21, 2017	May 2, 2017	May 2, 2017
Harvest 1st cut, 2017	May 30, 2017	June 14, 2017	June 13, 2017	June 13, 2017
Apply urea, 2017	May 31, 2017	June 22, 2017	NA	July 26, 2017
Harvest 2nd cut, 2017	July 9, 2017	August 3, 2017	August 3, 2017	October 15, 2017
Apply urea, 2017	July 10, 2017	August 9, 2017	NA	NA
Harvest 3rd cut, 2017	September 14, 2017	September 25, 2017	October 12, 2017	NA
Collect residual growth, 2018	March 29, 2018	April 24, 2018	April 30, 2018	May 1, 2018
Soil samples collected	March 28, 2018	April 24, 2018	April 12, 2018	April 12, 2018
Fertilizer treatments, 2018	May 15, 2018	April 24, 2018	May 2, 2018	May 2, 2018
Se treatments, 2018	May 16, 2018	May 4, 2018	May 2, 2018	May 2, 2018
Harvest 1st cut, 2018	July 17, 2018	June 5, 2018	June 14, 2018	May 25, 2018
Apply urea, 2018	July 18, 2018	June 13, 2018	NA	June 1, 2018
Harvest 2nd cut, 2018	September 22, 2018	July 31, 2018	July 31, 2018	September 26, 2018
Apply urea, 2018	September 23, 2018	August 6, 2108	NA	NA
Harvest 3rd cut, 2018	NA	September 7, 2108	September 26, 2018	NA
Collect residual growth, 2019	March 2019	NA	May 14, 2019	May 14, 2019

NA, None applied or no harvest; Not available.

Fertilization of (N)PKS included 0, (N)PK, and (N)PK plus S. The same agronomic principles were applied to meet forage species requirements for (N)PK and (N)PKS at each location (Gardner et al., 1981; Gardner et al., 2000; Koenig et al., 2009; Horneck et al., 2011; Miller et al., 2013; Moore et al., 2019). Based on soil analyses (**Table 1**) and production potential for each site, recommended fertilizer amounts of (N)PK and S were applied using appropriate combinations of gypsum, ammonium sulfate, ammonium phosphate, and urea. Soil analyses were performed by Ag Source Laboratories (Umatilla, OR) at the beginning of each growing season in 2017 and 2018 (**Table 2**). Soils were sampled from 0 to 15 cm in Roseburg, and 0 to 30 cm in Terrebonne and Union. At Roseburg, fertilizer included 0 (none), NPK (40 kg N ha^-1^, 100 kg P_2_O_5_ ha^-1^, and 100 kg K_2_O ha^-1^), and NPKS (NPK plus 34 kg S ha^-1^). Fertilizer was applied one day before Se application each year. In addition, urea (50 kg N ha^-1^) was applied to the treated plots after the first and second cuts in 2017, and after the first cut in 2018. At Terrebonne, fertilizer included 0 (none), NPK (134.5 kg N ha^-1^, 33.6 kg P_2_O_5_ ha^-1^, and 112 kg K_2_O ha^-1^), and NPKS (NPK plus 33.6 kg S ha^-1^). Fertilizer was applied on the day of Se application in 2017 and ten days before Se application in 2018. NutriSphere-N^®^-coated urea was applied after the first cut (134.5 kg N ha^-1^) and after the second cut (67.25 kg N ha^-1^). At Union, fertilizer included 0 (none), NPK (72.0 kg N ha^-1^, 34.0 kg P_2_O_5_ ha^-1^, and 192.0 kg K_2_O ha^-1^), and NPKS (NPK plus 12.0 kg S ha^-1^). Fertilizer was applied three days before Se application in 2017 and on the day of Se application in 2018. Urea (72.0 kg N ha^-1^) was applied to grasses after the first cut each year. For alfalfa, N was not applied.

Forages were harvested based on the recommended maturity stage (Collins et al., 2018); alfalfa was harvested at 10% bloom stage, and grass-dominated forages were harvested at early-anthesis stage on first cut and vegetative stage on the following cuts. At Roseburg, plots were harvested three times (25, 65, and 114 days post Se application) in year one, and twice (63 and 128 days post Se application) during year two, the latter due to a dry spring with slow forage growth (**Table 2**). Plots were harvested by a 0.9 m wide cycle-bar mower with a cutting height of 5 cm. At Terrebonne, plots were harvested 54, 104, and 157 days post Se application in year one and 32, 88, and 128 days post Se application in year two. Plots were harvested by a 1.1 m wide small plot sickle bar mower with a cutting height of 10 cm. At Union, alfalfa plots were harvested three times (42, 93, and 163 days post Se application) during year one and three times (43, 90, and 147 days post Se application) during year two, and grass plots were harvested twice (42 and 166 days post Se application) during year one and twice (23 and 147 days post Se application) during year two. Plots were harvested by a 0.9 m wide flail type harvester with a cutting height of 7.6 cm.

At the beginning of the new growing season, residual growth (i.e., regrowth before Se application) was collected on March 29, 2018 and March 30, 2019 in Roseburg. At Union, residual growth was clipped on April 30 and May 1, 2018 for alfalfa and grasses, respectively, and May 14, 2019. At Terrebonne, residual growth was clipped on April 15, 2018. In 2019, the integrity of the plots was not discernable to sample residual growth. The fields at Terrebonne and Union were utilized from October until mid-April as beef cattle pastures and the plots in Roseburg were mowed and the forage removed in the second half of October and late March. At the beginning of a new growing season, manure piles, if present, were removed.

### 2.2 Laboratory analytical methods

Representative forage grab samples (20 per plot in Roseburg and Union) were collected at each harvest time. We did not adjust for plant species differences. Forage samples were dried within hours of collection for at least 48 hours or until they reached a constant dry weight at 65°C. Samples were then ground with a Wiley mill with a 1.0 mm screen. At Terrebonne, 4 representative grab samples from each plot totaling 300-450 grams of forage were placed into Super 12 U-line paper bags and weighed within 10 minutes of collection with a portable Scout electronic scale to determine moist-sample weights in the field. The samples were then transported to a forage dryer and dried at 65°C until there was no longer any change in weight (approximately 3 days). Samples were then reweighed to calculate percent dry matter. Annual forage yield (kg forage DM ha^-1^) was determined by multiplying forage yield on a wet basis (kg forage ha^-1^) by percent dry matter.

The ground forage samples (approximately 50 g) were sent to the Utah Veterinary Diagnostic Laboratory (Logan, UT) to measure total Se concentration. As previously described (Davis et al., 2012), forage samples were prepared for Se analysis and Se was determined using inductively coupled argon plasma emission spectroscopy (ICP-MS; ELAN 6000, Perkin Elmer, Shelton, CT). Quantification of Se was performed by the standard addition method, using a 4-point standard curve. A quality-control sample (in similar matrix) was analyzed after every 5 samples, and analysis was considered acceptable if the Se concentration of the quality-control sample fell within ± 5% of the standard/reference value.

Ground forage samples (approximately 50 g) were sent to the Soil Health Laboratory at Oregon State University. Total carbon (C), nitrogen (N), and sulfate-sulfur (S) concentrations were determined by dry combustion using an Elementar vario macro cube (Elementar, Hanau, Germany). Forage samples were wrapped in aluminum foil and dropped into the analyzer through a blank-free helium-purged ball valve, and oxygen was injected over the sample at 1150°C. Separation of combustion gases was performed using a thermal desorption purge and trap chromatographic method (Bernius et al., 2014). The combustion gas components CO_2_ and SO_2_ were adsorbed onto two specific columns. Nitrogen flowed directly to a thermal conductivity detector. Based on the detector reading, gas components were released sequentially from their individual adsorption/desorption columns. Total time for analysis for carbon, N, and S was 10 minutes/sample.

### 2.3 Statistical analysis

The experimental design was a split-plot design with repeated measures at four forage-site combinations. Each split plot was replicated in triplicate. The whole plot was selenate amendment rate and the split-plot was fertilizer type. There were three replicates for each combination of amendment rate and fertilizer type.

Agronomic efficiencies were calculated as follows. For Se agronomic efficiency (%) = [(Se yield with selenate amendment – Se yield without selenate amendment)/amount of selenate amended] x 100. For N agronomic efficiency (%) = [(N yield with NPK(S) fertilization – N yield without NPK fertilization)/amount of N applied] x 100. For S agronomic efficiency (%) = [(S yield with (N)PKS application – S yield with (N)PK application)/amount of S applied] x 100.

The data were analysed as intention-to-treat analysis in PROC MIXED in SAS version 9.4 (SAS, 2009). Fixed effects in the model were foliar springtime selenate amendment rate (0, 45, or 90 g Se ha^-1^), the fertilizer type (none, (N)PK, or (N)PKS), and their interaction. The random effect was replicate (1, 2, or 3). Data are shown in **Tables 3**–**5** as least-squared means (LSM) and a pooled standard error of differences (SED). To determine LSM and SED, the statistical model was run for each forage/site × year combination separately. The *P*-values in the tables refer to those of the fixed effects in the model.

**Table 3:** Effect of springtime sodium-selenate foliar application rate (0, 45, and 90 g Se ha^-1^) and nitrogen-phosphorus-potassium-sulfur fertilization (none, (N)PK, or (N)PKS) on forage yield (kg ha^-1^ DM) and forage Se concentration, yield, and agronomic efficiency in forages across Oregon^1^.

Se-Application Rate	None			(N)PK			(N)PKS					P-values			
	0	45	90	0	45	90	0	45	90	SED		Se		Fertilizer	Se х Fert
Year & Forage Type	**Annual Forage Yield (kg forage DM ha^-1^)**														
2017															
Alfalfa^2^	10505	10671	11107	12603	12087	12782	11999	12570	11743	546		0.91		<0.0001	0.18
Grasses^2^	2090	3548	1876	3981	3608	3592	4857	4582	3596	1027		0.55		0.005	0.40
Orchard Grass^3^	3491	4354	3479	10507	10155	9711	11838	11659	11769	773		0.80		<0.0001	0.53
Grass-Clover^4^	3360	3253	3936	5416	5369	5189	5233	5435	5462	453		0.80		<0.0001	0.53
2018															
Alfalfa	12197	11663	11395	14092	13953	15118	14888	13379	13869	771		0.31		0.0001	0.35
Grasses	1472	3807	2203	5004	4712	4459	4980	4712	3978	1483		0.87		0.01	0.30
Orchard Grass	4028	4894	4158	9718	8947	10561	14881	14436	14468	949		0.93		<0.0001	0.096
Grass-Clover	6493	5799	6645	7553	7540	8111	7768	8493	8284	1143		0.89		0.002	0.70
	**Average Annual Se Concentration (mg Se kg^-1^ forage DM)**														
2017															
Alfalfa	0.10^c^	1.46^b^	2.93^a^	0.13^c^	1.44^b^	2.76^a^	0.07^c^	1.22^b^	2.71^a^	0.28		<0.0001		0.45	0.92
Grasses	0.11^c^	1.51^b^	2.61^a^	0.09^c^	1.51^b^	2.58^a^	0.07^c^	1.32^b^	3.21^a^	0.25		<0.0001		0.57	0.16
Orchard Grass	0.08^c^	1.13^b^	2.88^a^	0.07^c^	1.02^b^	2.11^a^	0.08^c^	1.13^b^	2.41^a^	0.26		<0.0001		0.10	0.26
Grass-Clover	0.08^c^	1.30^b^	2.58^a^	0.09^c^	1.15^b^	2.52^a^	0.09^c^	1.19^b^	2.48^a^	0.46		0.003		0.84	0.99
2018															
Alfalfa	0.18^c^	1.35^b^	1.98^a^	0.20^c^	1.06^b^	2.00^a^	0.16^c^	1.03^b^	1.81^a^	0.25		<0.0001		0.32	0.69
Grasses	0.13^c^	1.50^b^	3.02^a^	0.12^c^	1.69^b^	3.60^a^	0.14^c^	1.45^b^	3.81^a^	0.46		<0.0001		0.52	0.64
Orchard Grass	0.10^c^	1.25^b^	2.65^a^	0.11^c^	0.83^b^	2.03^a^	0.04^c^	1.18^b^	2.32^a^	0.24		<0.0001		0.09	0.44
Grass-Clover	0.09^c^	0.86^b^	1.30^a^	0.09^c^	0.67^b^	1.29^a^	0.10^c^	0.72^b^	1.18^a^	0.11		<0.0001		0.39	0.63
	**Annual Se Harvested (g Se ha^-1^)**														
2017															
Alfalfa	1.07^c^	15.55^b^	32.37^a^	1.60^c^	17.44^b^	35.45^a^	0.89^c^	15.31^b^	31.73^a^	3.39		<0.0001		0.35	0.94
Grasses	0.21^b^	5.59^a^	4.82^a^	0.37^b^	5.43^a^	9.24^a^	0.36^c^	6.09^b^	11.57^a^	2.12		0.009		0.045	0.053
Orchard Grass	0.29^b^	5.26^ab^	10.03^a^	0.70^c^	10.60^b^	20.49^a^	0.93^c^	13.33^b^	28.32^a^	2.84		0.0003		0.0001	0.005
Grass-Clover	0.28^b^	4.30^b^	10.36^a^	0.45^c^	6.16^b^	12.86^a^	0.44^c^	6.30^b^	13.60^a^	2.26		0.004		0.02	0.33
2018															
Alfalfa	2.23^c^	15.76^b^	22.72^a^	2.79^c^	14.83^b^	29.99^a^	2.31^c^	14.03^b^	25.02^a^	3.54		0.0004		0.31	0.32
Grasses	0.18^b^	3.92^ab^	6.65^a^	0.61^c^	7.15^b^	15.63^a^	0.71^c^	6.64^b^	15.12^a^	2.53		0.0004		0.03	0.20
Orchard Grass	0.41^b^	6.16^ab^	10.93^a^	1.09^b^	7.49^b^	21.77^a^	0.55^c^	17.07^b^	33.51^a^	3.26		0.0004		<0.0001	0.001
Grass-Clover	0.61^c^	5.01^b^	8.66^a^	0.72^c^	5.07^b^	10.41^a^	0.75^c^	6.22^b^	9.72^a^	1.15		0.0004		0.09	0.17
	**Se Agronomic Efficiency (%)^5^**														
2017															
Alfalfa	Ref.^6^	32.17	34.77	Ref.	35.21	37.61	Ref.	32.04	34.61	5.20		0.55		0.56	0.99
Grasses	Ref.	11.96	5.12	Ref.	11.24	9.86	Ref.	12.74	12.46	4.46		0.47		0.27	0.36
Orchard Grass	Ref.	11.06	10.83	Ref.	22.01	22.00	Ref.	27.55	30.43	6.15		0.85		0.003	0.89
Grass-Clover	Ref.	8.93	11.19	Ref.	12.69	13.78	Ref.	13.01	14.61	3.71		0.66		0.02	0.88
2018															
Alfalfa	Ref.	30.06	22.76	Ref.	26.76	30.22	Ref.	26.05	25.23	7.67		0.83		0.66	0.28
Grasses	Ref.	8.32	7.18	Ref.	14.52	16.68	Ref.	13.16	16.00	5.05		0.70		0.10	0.83
Orchard Grass	Ref.	12.79	11.69	Ref.	14.22	22.98	Ref.	36.70	36.63	6.09		0.63		0.0002	0.30
Grass-Clover	Ref.	9.78	8.93	Ref.	9.68	10.76	Ref.	12.17	9.96	2.64		0.79		0.31	0.33

^1^Data are shown as least-squared means (LSMs) of sodium selenate application rates within fertilization types with a pooled standard error of difference (SED) for each year. Forages were harvested 2 times (grasses both years and grass-clover in 2018) or 3 times (alfalfa and orchard grass both years and grass-clover in 2017) per year. LSMs of forages within fertilizer type (none, (N)PK, or (N)PKS) with different lower-case superscripts are significantly different from each other (P≤0.05). LSMs of forages within fertilizer type (none, (N)PK, or (N)PKS) with the same lower-case superscripts or no superscripts are not significantly different from each other (P≥0.05).

^2^For alfalfa and grasses at Union (eastern Oregon), sodium selenate (RETORTE Ulrich Scharrer GmbH, Röthenbach, Germany) was applied May 2, 2017 and 2018. Fertilizer was applied three days before selenate application in 2017 and on the day of selenate application in 2018 and included for alfalfa 0 (none), PK (34.0 kg P_2_O_5_ ha^-1^, and 192.0 kg K_2_O ha^-1^), and PKS (PK plus 12.0 kg S ha^-1^) and for grasses 0 (none), NPK (72.0 kg N ha^-1^, 34.0 kg P_2_O_5_ ha^-1^, and 192.0 kg K_2_O ha^-1^), and NPKS (NPK plus 12.0 kg S ha^-1^). In addition, urea (72.0 kg N ha^-1^) was applied after the first grass cut.

^3^For orchard grass at Terrebonne (central Oregon), sodium selenate was applied April 21, 2017 and May 4, 2018. Fertilizer was applied on the day of selenate application in 2017 and ten days before selenate application in 2018 and included 0 (none), NPK (134.5 kg N ha^-1^, 33.6 kg P_2_O_5_ ha^-1^, and 112 kg K_2_O ha^-1^), and NPKS (NPK plus 33.6 kg S ha^-1^). In addition, NutriSphere-N^®^-coated urea was applied after the first and second cut (134.5 kg N ha^-1^) and after the third cut (67.25 kg N ha^-1^).

^4^For grass-clover, sodium selenate was applied May 5, 2017 and May 16, 2018. One day before Se application, fertilizer was applied including 0 (none), NPK (40 kg N ha^-1^, 100 kg P_2_O_5_ ha^-1^, and 100 kg K_2_O ha^-1^), and NPKS (NPK plus 34 kg S ha^-1^). In addition, urea (50 kg N ha^-1^) was applied to the treated plots after each harvest.

^5^Selenium agronomic efficiency (%) = [(Se yield with selenate amendment – Se yield without selenate amendment)/amount of selenate amended] x 100.

^6^Ref. is designated as the unexposed control group (0 g Se ha^-1^).

**Table 4:** Effect of springtime sodium-selenate foliar application rate (0, 45, and 90 g Se ha^-1^) and nitrogen-phosphorus-potassium-sulfur fertilization (none, (N)PK, or (N)PKS) on annual forage N concentration, yield, and agronomic efficiency in forages across Oregon^1^.

Se-Application Rate	None			(N)PK			(N)PKS				P-values		
	0	45	90	0	45	90	0	45	90	SED	Se	Fertilizer	Se х Fert
Year & Forage Type	**Average Annual N Concentration (forage DM %)**												
2017													
Alfalfa^2^	2.84	2.88	2.79	2.82	2.85	2.90	2.97	2.89	2.93	0.07	0.99	0.06	0.28
Grasses^2^	1.51	1.64	1.40	1.72	1.70	1.73	1.72	1.65	1.65	0.14	0.74	0.06	0.61
Orchard Grass^3^	1.41	1.45	1.39	1.83	1.80	1.88	1.76	1.73	1.72	0.05	0.94	<0.0001	0.38
Grass-Clover^4^	2.43	2.37	2.21	2.45	2.38	2.47	2.57	2.48	2.39	0.17	0.71	0.056	0.28
2018													
Alfalfa	2.61	2.65	2.68	2.74	2.68	2.62	2.74	2.68	2.72	0.12	0.90	0.53	0.75
Grasses	1.71	1.76	1.78	2.06	1.98	1.92	2.05	1.96	2.08	0.15	0.91	0.02	0.80
Orchard Grass	1.28	1.34	1.34	2.16^b^	2.26^ab^	2.39^a^	1.96	2.10	1.96	0.09	0.30	<0.0001	0.097
Grass-Clover	2.23	2.27	2.26	2.44	2.29	2.30	2.44	2.29	2.23	0.19	0.93	0.71	0.73
	**Annual N Harvested (kg N ha^-1^)**												
2017													
Alfalfa	299.0	307.6	310.5	354.8	343.8	369.7	356.4	362.5	343.5	13.87	0.76	0.0001	0.35
Grasses	31.67	65.15	26.42	68.68	61.59	62.42	83.91	76.27	60.20	22.37	0.58	0.02	0.41
Orchard Grass	48.87	62.84	48.37	192.4	182.5	182.2	208.6	201.7	202.9	13.44	0.88	<0.0001	0.40
Grass-Clover	81.43	77.03	87.54	133.5	128.0	129.0	135.0	134.7	130.3	14.65	0.96	<0.0001	0.88
2018													
Alfalfa	318.1	309.3	305.6	385.6	367.2	397.0	407.2^a^	357.4^b^	376.2^ab^	21.86	0.16	0.0002	0.56
Grasses	24.84	76.63	39.38	102.9	80.98	85.90	101.6	91.51	83.62	33.25	0.88	0.01	0.35
Orchard Grass	51.68	65.90	55.35	209.1^b^	199.6^b^	251.6^a^	291.5	303.0	283.4	14.34	0.54	<0.0001	0.007
Grass-Clover	145.8	131.7	151.7	171.3	172.4	185.8	190.1	189.5	184.6	27.27	0.92	0.003	0.84
	**Nitrogen Agronomic Efficiency (%)^5^**												
2017													
Alfalfa	NA^6^	NA	NA	NA	NA	NA	NA	NA	NA	NA	NA	NA	NA
Grasses	Ref.^6^	Ref.	Ref.	25.70	23.27	25.01	36.28	33.46	23.46	11.42	0.79	0.24	0.55
Orchard Grass	Ref.	Ref.	Ref.	35.58	29.65	33.16	39.58	34.41	38.30	3.70	0.31	0.01	0.93
Grass-Clover	Ref.	Ref.	Ref.	37.19	36.40	29.39	38.25	41.21	30.56	11.54	0.65	0.63	0.93
2018													
Alfalfa	NA^6^	NA	NA	NA	NA	NA	NA	NA	NA	NA	NA	NA	NA
Grasses	Ref.^6^	Ref.	Ref.	54.19	41.41	32.30	53.31	48.72	30.72	17.34	0.40	0.81	0.83
Orchard Grass	Ref.	Ref.	Ref.	39.01^ab^	33.13^b^	48.63^a^	59.43	58.77	56.52	4.22	0.24	<0.0001	0.02
Grass-Clover	Ref.	Ref.	Ref.	18.17	29.10	24.38	31.60	41.29	23.47	18.71	0.71	0.42	0.80

^1^Data are shown as least-squared means (LSMs) of sodium selenate application rates within fertilization types with a pooled standard error of difference (SED) for each year. Forages were harvested 2 times (grasses both years and grass-clover in 2018) or 3 times (alfalfa and orchard grass both years and grass-clover in 2017) per year. LSMs of forages within fertilizer type (none, (N)PK, or (N)PKS) with different lower-case superscripts are significantly different from each other (P≤0.05). LSMs of forages within fertilizer type (none, (N)PK, or (N)PKS) with the same lower-case superscripts or no superscripts are not significantly different from each other (P≥0.05).

^2^For alfalfa and grasses at Union (eastern Oregon), sodium selenate (RETORTE Ulrich Scharrer GmbH, Röthenbach, Germany) was applied May 2, 2017 and 2018. Fertilizer was applied three days before selenate application in 2017 and on the day of selenate application in 2018 and included for alfalfa 0 (none), PK (34.0 kg P_2_O_5_ ha^-1^, and 192.0 kg K_2_O ha^-1^), and PKS (PK plus 12.0 kg S ha^-1^) and for grasses 0 (none), NPK (72.0 kg N ha^-1^, 34.0 kg P_2_O_5_ ha^-1^, and 192.0 kg K_2_O ha^-1^), and NPKS (NPK plus 12.0 kg S ha^-1^). In addition, urea (72.0 kg N ha^-1^) was applied after the first grass cut.

^3^For orchard grass at Terrebonne (central Oregon), sodium selenate was applied April 21, 2017 and May 4, 2018. Fertilizer was applied on the day of selenate application in 2017 and ten days before selenate application in 2018 and included 0 (none), NPK (134.5 kg N ha^-1^, 33.6 kg P_2_O_5_ ha^-1^, and 112 kg K_2_O ha^-1^), and NPKS (NPK plus 33.6 kg S ha^-1^). In addition, NutriSphere-N^®^-coated urea was applied after the first and second cut (134.5 kg N ha^-1^) and after the third cut (67.25 kg N ha^-1^).

^4^For grass-clover, sodium selenate was applied May 5, 2017 and May 16, 2018. One day before Se application, fertilizer was applied including 0 (none), NPK (40 kg N ha^-1^, 100 kg P_2_O_5_ ha^-1^, and 100 kg K_2_O ha^-1^), and NPKS (NPK plus 34 kg S ha^-1^). In addition, urea (50 kg N ha^-1^) was applied to the treated plots after each harvest.

^5^Nitrogen agronomic efficiency (%) = [(N yield with NPK(S) application – N yield without NPK application)/amount of N applied] x 100.

^6^Ref. is designated as the unexposed control group: none. NA, not applicable.

**Table 5:** Effect of springtime sodium-selenate foliar application rate (0, 45, and 90 g Se ha^-1^) and nitrogen-phosphorus-potassium-sulfur fertilization (none, (N)PK, or (N)PKS) on annual forage S concentration, yield, and agronomic efficiency in forages across Oregon^1^.

Se-Application Rate	None			(N)PK			(N)PKS				P-values			
	0	45	90	0	45	90	0	45	90	SED	Se	Fertilizer		Se х Fert
Year & Forage Type	**Average Annual S Concentration (forage DM %)**													
2017														
Alfalfa^2^	0.210	0.210	0.230	0.250	0.277	0.260	0.263	0.250	0.260	0.013	0.73	<0.0001		0.02
Grasses^2^	0.190	0.183	0.183	0.203	0.193	0.213	0.193^a^	0.167^b^	0.180^ab^	0.011	0.31	0.001		0.23
Orchard Grass^3^	0.170	0.170	0.173	0.153	0.150	0.157	0.193	0.187	0.180	0.011	0.90	0.0004		0.75
Grass-Clover^4^	0.307	0.310	0.283	0.333	0.303	0.333	0.330	0.337	0.320	0.018	0.74	0.005		0.069
2018														
Alfalfa	0.200	0.200	0.207	0.240	0.220	0.230	0.260	0.240	0.240	0.012	0.44	<0.0001		0.25
Grasses	0.170	0.167	0.160	0.183	0.167	0.180	0.180	0.193	0.190	0.010	0.93	0.006		0.23
Orchard Grass	0.167	0.173	0.170	0.133	0.133	0.143	0.157	0.160	0.157	0.007	0.63	<0.0001		0.53
Grass-Clover	0.297	0.313	0.330	0.293	0.307	0.307	0.307	0.317	0.330	0.019	0.30	0.30		0.92
	**Annual S Harvested (kg S ha^-1^)**													
2017														
Alfalfa	22.02	22.83	25.22	31.70	33.34	33.18	31.57	31.57	30.74	1.88	0.57	<0.0001		0.55
Grasses	3.92	6.40	3.43	8.11	7.05	7.62	9.19	7.58	6.50	1.81	0.62	0.004		0.26
Orchard Grass	5.85	7.46	6.06	15.98	15.30	15.17	22.78	21.88	21.23	1.42	0.79	<0.0001		0.35
Grass-Clover	10.21	10.12	11.24	17.72	16.27	17.28	17.27	18.38	17.56	1.40	0.91	<0.0001		0.55
2018														
Alfalfa	24.53	23.35	23.27	33.98^ab^	30.58^b^	34.87^a^	38.25^a^	31.91^b^	33.47^b^	1.68	0.09	<0.0001		0.02
Grasses	2.54	6.34	3.56	8.99	6.64	7.88	8.82	8.85	7.47	2.41	0.87	0.005		0.30
Orchard Grass	6.70	8.54	7.09	13.08	11.84	15.13	23.20	23.38	22.52	1.57	0.91	<0.0001		0.02
Grass-Clover	19.26	18.20	22.38	22.01	23.11	24.92	23.82	26.75	27.67	4.14	0.66	0.005		0.84
	**Sulfur Agronomic Efficiency (%)^5^**													
2017														
Alfalfa	NA^6^	NA	NA	Ref.^6^	Ref.	Ref.	-1.12	-14.75	-20.33	14.73	0.45	NA		NA
Grasses	NA	NA	NA	Ref.	Ref.	Ref.	8.98	4.42	-9.29	11.98	0.35	NA		NA
Orchard Grass	NA	NA	NA	Ref.	Ref.	Ref.	20.25	19.59	18.06	4.63	0.89	NA		NA
Grass-Clover	NA	NA	NA	Ref.	Ref.	Ref.	-0.88	5.08	3.16	4.95	0.51	NA		NA
2018														
Alfalfa	NA^6^	NA	NA	Ref.^6^	Ref.	Ref.	35.55^a^	11.13^b^	-11.73^c^	9.11	0.006	NA		NA
Grasses	NA	NA	NA	Ref.	Ref.	Ref.	-1.46	18.42	-3.37	10.82	0.16	NA		NA
Orchard Grass	NA	NA	NA	Ref.	Ref.	Ref.	30.14	34.34	22.00	5.87	0.18	NA		NA
Grass-Clover	NA	NA	NA	Ref.	Ref.	Ref.	6.65	7.14	5.45	15.47	0.99	NA		NA

^1^Data are shown as least-squared means (LSMs) of sodium selenate application rates within fertilization types with a pooled standard error of difference (SED) for each year. Forages were harvested 2 times (grasses both years and grass-clover in 2018) or 3 times (alfalfa and orchard grass both years and grass-clover in 2017) per year. LSMs of forages within fertilizer type (none, (N)PK, or (N)PKS) with different lower-case superscripts are significantly different from each other (P≤0.05). LSMs of forages within fertilizer type (none, (N)PK, or (N)PKS) with the same lower-case superscripts or no superscripts are not significantly different from each other (P≥0.05).

^2^For alfalfa and grasses at Union (eastern Oregon), sodium selenate (RETORTE Ulrich Scharrer GmbH, Röthenbach, Germany) was applied May 2, 2017 and 2018. Fertilizer was applied three days before selenate application in 2017 and on the day of selenate application in 2018 and included for alfalfa 0 (none), PK (34.0 kg P_2_O_5_ ha^-1^, and 192.0 kg K_2_O ha^-1^), and PKS (PK plus 12.0 kg S ha^-1^) and for grasses 0 (none), NPK (72.0 kg N ha^-1^, 34.0 kg P_2_O_5_ ha^-1^, and 192.0 kg K_2_O ha^-1^), and NPKS (NPK plus 12.0 kg S ha^-1^). In addition, urea (72.0 kg N ha^-1^) was applied after the first grass cut.

^3^For orchard grass at Terrebonne (central Oregon), sodium selenate was applied April 21, 2017 and May 4, 2018. Fertilizer was applied on the day of selenate application in 2017 and ten days before selenate application in 2018 and included 0 (none), NPK (134.5 kg N ha^-1^, 33.6 kg P_2_O_5_ ha^-1^, and 112 kg K_2_O ha^-1^), and NPKS (NPK plus 33.6 kg S ha^-1^). In addition, NutriSphere-N^®^-coated urea was applied after the first and second cut (134.5 kg N ha^-1^) and after the third cut (67.25 kg N ha^-1^).

^4^For grass-clover, sodium selenate was applied May 5, 2017 and May 16, 2018. One day before Se application, fertilizer was applied including 0 (none), NPK (40 kg N ha^-1^, 100 kg P_2_O_5_ ha^-1^, and 100 kg K_2_O ha^-1^), and NPKS (NPK plus 34 kg S ha^-1^). In addition, urea (50 kg N ha^-1^) was applied to the treated plots after each harvest.

^5^Sulfur agronomic efficiency (%) = [(S yield with (N)PKS application – S yield with (N)PK application)/amount of S applied] x 100.

^6^Ref. is designated as the unexposed control group: (N)PK. NA, not applicable.

Using ESTIMATE statements, the linear effect of selenate amendment rate was calculated by comparing results of 90 g Se ha^-1^ amended plots with those of 0 g Se ha^-1^ amended plots; the non-linear or lack of linearity effect of selenate amendment rate was calculated by comparing results for 45 g Se ha^-1^ amended plots with those of 0 and 90 g Se ha^-1^ amended plots combined; the effect of (N)PK-fertilization was calculated by comparing results for (N)PK-fertilized with non-fertilized plots; and the effect of S-fertilization was calculated by comparing results for (N)PKS-fertilized with (N)PK-fertilized plots. All contrasts were orthogonal. The *P*-values in the text refer to those calculated by the ESTIMATE statements. PROC CORR was used to calculate Pearson’s correlation coefficients. All statistical tests were two-sided. Statistical significance was declared at *p* ≤ 0.05 and a statistical trend was declared at 0.05 < *p* ≤ 0.10.

## 3 Results

### 3.1 Response of forages to different selenate application rates

**Figure 3:**
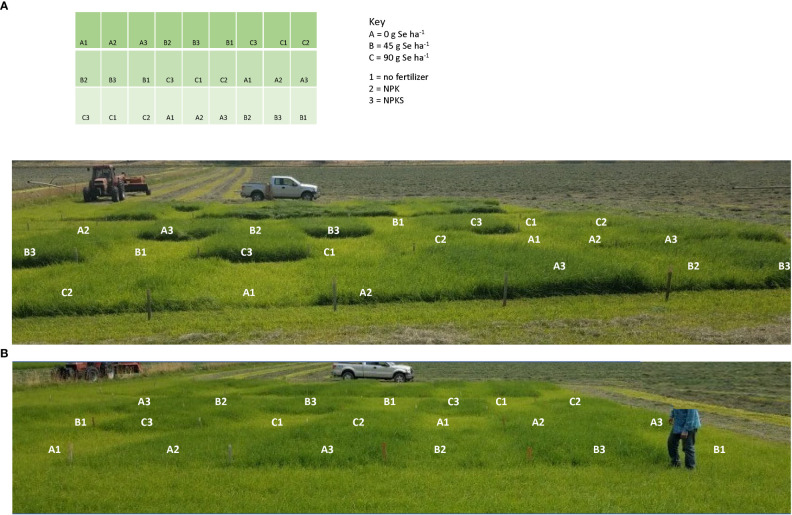
Forage plots at Terrebonne (central Oregon) in 2018 showing early growth of **(A)** second cutting, and **(B)** third cutting. Sodium selenate was applied to plots at rates of 0, 45, and 90 g Se ha^-1^ on May 4, 2018. Fertilizer was applied ten days prior to Se application, and consisted of no fertilizer, NPK (134.5 kg N ha^-1^, 33.6 kg P_2_O_5_ ha^-1^, and 112 kg K_2_O ha^-1^), or NPKS (NPK plus 33.6 kg S ha^-1^). Forage growth shows the positive effects of S fertilization, whereas selenate amendment did not affect forage yields.

Foliar springtime selenate amendment at 45 and 90 g Se ha^-1^ did not affect annual forage yields (kg forage DM ha^-1^) in Oregon in 2017 and 2018 (all main effects *P* > 0.30; **Table 3**; **Figure 3**). Soil Se concentrations were low at all three sites: Roseburg (0.18 mg Se kg^-1^ soil), Terrebonne (0.12 mg Se kg^-1^ soil), and Union (0.15 mg Se kg^-1^ soil). Plant Se concentrations without selenate amendment were lower than soil Se concentrations: Roseburg (0.08-0.10 mg Se kg^-1^ forage DM), Terrebonne (0.04-0.11 mg Se kg^-1^ forage), and Union (0.07-0.20 mg Se kg^-1^ forage with lower concentrations in 2017 than in 2018). Without Se-amendment, alfalfa had higher Se concentrations (2018) and yields (2017 and 2018) than grasses at Union. After Se-amendment, alfalfa had lower Se concentrations (2018) than grasses.

**Figure 4:**
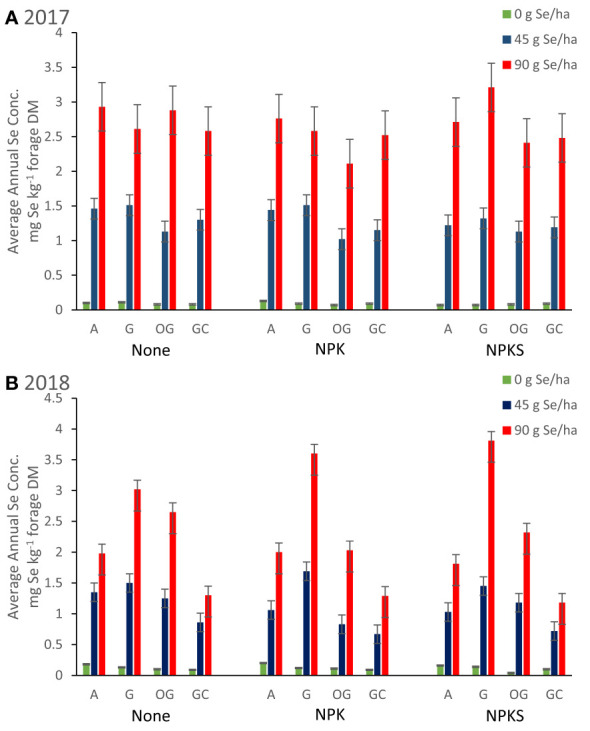
Average annual Se concentrations in forages (mg Se kg^-1^ forage DM) for plots in Union (eastern Oregon; alfalfa (A), and grass (G) mixture), Terrebonne (central Oregon; orchard grass, OG), and Roseburg (western Oregon; grass-clover mixture, GC) in **(A)** 2017 and **(B)** 2018. Sodium selenate was applied to plots at rates of 0, 45, or 90 g Se ha^-1^. Fertilizer was applied as none, (N)PK, or (N)PKS at approximately the same time. Selenate amendment linearly increased forage Se concentrations (all *P* < 0.004), and were highly correlated to annual Se yield (g Se ha^-1^; r = +0.70; *P* < 0.0001). Co-application of selenate and (N)PK decreased forage Se concentrations in S-deficient forage sites (Terrebonne; both Se amendment rates *P* < 0.04), but not at the other two forage sites (all *P* > 0.19). Sulfate-S application decreased forage Se concentrations, when plant availability of Se was low (0 g Se ha^-1^; Terrebonne in 2018).

Selenate amendment at 45 and 90 g Se ha^-1^ increased average Se concentrations (mg Se kg^-1^ forage) and annual Se yield (g Se ha^-1^) of the harvested forage (**Table 3**; **Figure 4**). The effect was significant at both amendment rates for all combinations of forage species, locations and years (all main effects *P* < 0.004). Selenate amendment linearly increased forage Se concentrations (linearity: all *P* < 0.004; lack of linearity: all *P* > 0.31) and annual Se yield (linearity: all *P* < 0.002; lack of linearity: all *P* > 0.42). Forage Se concentrations were highly correlated to annual Se yield (r = +0.70; *P* < 0.0001), but not to yields and concentrations of N or S (all *P* > 0.03). Among Se-amended plots, grasses at Union in 2018 had the highest Se concentration, whereas grass-clover at Roseburg in 2018 had the lowest.

Selenium agronomic efficiency (%) after selenate amendment, calculated by the formula [(Se yield with selenate amendment – Se yield without selenate amendment)/amount of selenate amended] x 100, was strongly correlated to annual forage yield (r = +0.81; *P* < 0.0001) and was not affected by selenate amendment rate (all *P* > 0.47). Alfalfa at Union and NPKS-fertilized orchard grass at Terrebonne had the highest Se agronomic efficiencies (25 to 37%), whereas grasses at Union and grass-clover at Roseburg had the lowest (5 to 17%). Similarly, forage Se concentrations and annual Se yields doubled from the 45 to the 90 g Se ha^-1^ amendment rate in high yielding forages (i.e., alfalfa at Union and orchard grass at Terrebonne) and less than doubled in low yielding forages (i.e. grass-clover at Roseburg in 2018 and not-fertilized and NPK-fertilized grasses at Union in 2017).

Selenium agronomic efficiency depended on the efficient conversion of N and Se into plant proteins. In support, Se agronomic efficiency (%) was strongly correlated to forage yield of Se (r = +0.85; *P* < 0.0001), forage N yield (r = +0.78; *P* < 0.0001), and N agronomic efficiency (r = +0.55; *P* < 0.0001; calculated by the formula [(N yield with NPK(S) fertilization – N yield without NPK fertilization)/amount of N applied] x 100), and forage nitrogen concentration (r = +0.49; *P* < 0.0001).

Foliar springtime selenate amendment at 45 and 90 g Se ha^-1^ did not affect annual forage N concentrations (all *P* > 0.19), N yields (all but one main effect *P* > 0.28), and N agronomic efficiencies (%) after N fertilization in Oregon in both years for all combinations of forages, sites, and S application rates (all main effects *P* > 0.15; **Table 4**).

Foliar springtime selenate amendment at 45 and 90 g Se ha^-1^ did not decrease forage S concentrations (all *P* > 0.14) nor S yields (all but one main effect *P* > 0.30; **Table 5**). The only exception was S yields of alfalfa at Union in 2018, when forage S yields were lower at 45 g Se ha^-1^ compared with the two other amendment rates combined (*P* = 0.05).

### 3.2 Response of Se-amended forages to NPK fertilizer

In Oregon, 2018 was a good forage production year, whereas 2017 was a lower forage production year. Almost all fertilizer, Se amendment, and forage site/species combinations produced more forage biomass in 2018 than in 2017 (**Table 3**) because of warmer temperatures in May 2018 than in May 2017.Without fertilization, forage grass yields were very low. At Terrebonne and Union, selenate amendment at 45 g ha^-1^ increased forage yields of non-fertilized grass plots, but not among NPK-fertilized plots. There was a significant interaction between selenate amendment rate and NPK fertilization for orchard grass forage yield in 2018 (*P* = 0.02) and statistical tendencies for grasses forage yield in 2017 (*P* = 0.09) and 2018 (*P* = 0.07). Fertilization with NPK or PK (for alfalfa) increased annual forage yields (kg forage DM ha^-1^), when compared with no fertilization (all main effects *P* < 0.03). The greatest forage yield increases were observed for orchard grass at Terrebonne in central Oregon (+ 4,000 to 6,000 kg forage ha^-1^), whereas the other forage yield increases were similar in magnitude (+ 1,000 to 2,000 kg forage ha^-1^).

Fertilization with NPK alone did not affect forage Se concentrations (**Table 3**; **Figure 4**). Co-application of selenate and (N)PK decreased forage Se concentrations at Terrebonne (both Se amendment rates *P* < 0.04), but not at the other two forage sites (all *P* > 0.19). Fertilization with NPK increased forage Se yields of orchard grass at Terrebonne in 2017 (*P* = 0.002) and 2018 (*P* = 0.02), grasses at Union in 2018 (*P* = 0.02), and grass-clover at Roseburg in 2017 (*P* = 0.03) and 2018 (*P* = 0.09). The largest increases in forage Se yields were observed for orchard grass at Terrebonne, as both NPK-fertilization and each additional 45 g ha^-1^ Se amendment increased Se yield by 5 g Se ha^-1^ to a total of 20 g Se ha^-1^ in NPK-fertilized and 90 g ha^-1^ Se amended plots (*P* interaction = 0.01 in 2017 and 0.02 in 2018). Co-application of selenate and NPK increased Se agronomic efficiency of grasses at Union in 2018 (*P* = 0.05), grass-clover at Roseburg in 2017 (*P* = 0.03), and orchard grass at Terrebonne in 2017 (*P* = 0.02) and in 2018 (only for 90 g ha^-1^ Se amendment). Fertilization with PK did not affect alfalfa Se concentrations and yields; co-application of selenate and PK did not affect Se agronomic efficiency (all *P* > 0.17).

NPK fertilization increased forage N concentrations (forage DM %) at Union for grasses in 2017 (*P* = 0.03) and in 2018 (*P* = 0.02), at Terrebonne for orchard grass in 2017 and 2018 (both *P* < 0.0001), and at Roseburg for grass-clover in 2017 (*P* = 0.09; only for 90 g ha^-1^ Se amendment; **Table 4**). No effect of PK fertilization on forage nitrogen concentrations was observed for alfalfa at Union (*P* > 0.70).

**Figure 5:**
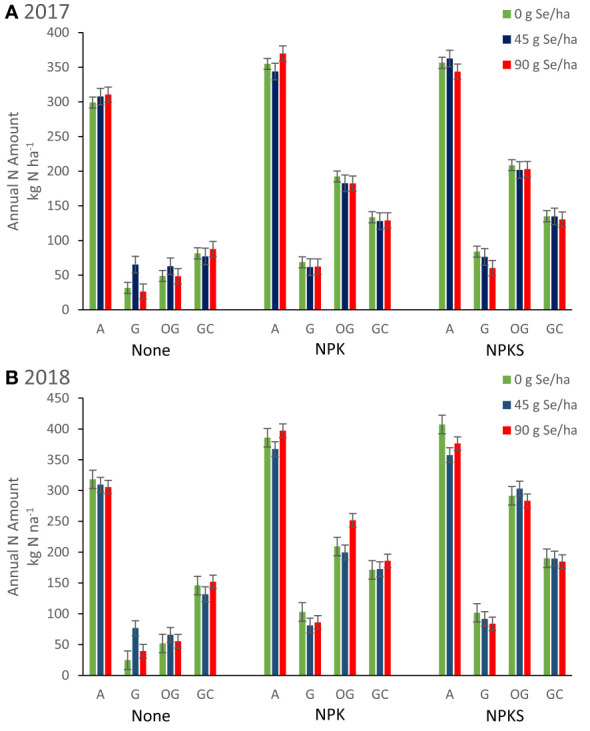
Annual nitrogen harvested from forages (kg N ha^-1^) for plots in Union (eastern Oregon; alfalfa (A), and grass (G) mixture), Terrebonne (central Oregon; orchard grass, OG), and Roseburg (western Oregon; grass-clover mixture, GC) in **(A)** 2017 and **(B)** 2018. Sodium selenate was applied to plots at rates of 0, 45, or 90 g Se ha^-1^. Fertilizer was applied as none, (N)PK, or (N)PKS at approximately the same time. Sulfur fertilization increased forage N yield for orchard grass at Terrebonne in 2017 (*P* = 0.004) and 2018 (*P* < 0.0001).

Fertilization with (N)PK increased annual forage nitrogen yields (kg N ha^-1^), when compared with no fertilization (all main effects *P* < 0.05; **Table 4**; **Figure 5**). At Terrebonne, co-application of NPK and 90 g ha^-1^ Se amendment increased N yields more compared with the two lower Se amendment rates (*P* interaction < 0.04). Nitrogen agronomic efficiencies were consistently positive (18-59%; **Table 4**). Compared with Se agronomic efficiencies, N agronomic efficiencies were higher and, with exception of orchard grass at Terrebonne, more variable than Se agronomic efficiencies (**Tables 3**, **4**). Both Se and N agronomic efficiencies depended on total forage yield (r = +0.45; *P <*0.0001). Orchard grass at Terrebonne in 2018 had the highest N agronomic efficiencies (33-49%), whereas grasses at Union in 2017 (23-26%) and grass-clover at Roseburg in 2018 (18-29**%**) had the lowest (**Table 4**).

The effect of (N)PK fertilization on forage S concentration depended on forage site and year (**Table 5**). At Terrebonne, NPK fertilization decreased forage S concentrations in both years, whereas forage S concentrations were increased at Union and at Roseburg in 2017. At all forage sites, NPK fertilization increased forage S yields (all main effects *P* < 0.04; **Table 5**). Co-application of NPK and 90 g Se ha^-1^ resulted in the highest S yields in 2018 at Terrebonne (*P* interaction = 0.005) and for alfalfa and grasses at Union (both *P* interactions = 0.05).

### 3.3 Response of Se-amended forages to NPK plus S fertilizer

**Figure 6:**
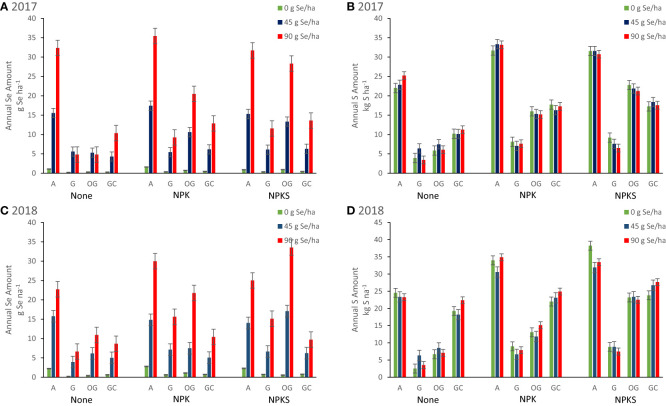
Annual Se (g Se ha^-1^) and S (kg S ha^-1^) harvested from forages in Union (eastern Oregon; alfalfa (A), and grass (G) mixture), Terrebonne (central Oregon; orchard grass, OG), and Roseburg (western Oregon; grass-clover mixture, GC) in **(A, B)** 2017, and **(C, D)** 2018, respectively. Sodium selenate was applied to plots at rates of 0, 45, or 90 g Se ha^-1^. Fertilizer was applied as none, (N)PK, or (N)PKS at approximately the same time. Sulfur fertilization without Se amendment increased S yield of alfalfa at Union (*P* interaction = 0.03). At Terrebonne, co-application of NPKS and selenate increased harvested Se yields in 2017 (*P* = 0.03) and even more so in 2018 (*P* = 0.0007). At Roseburg, higher annual S yield from GC in 2018 in the absence of S fertilization (S was present in the irrigation water) was associated with lower annual Se yield after selenate amendment.

With the exception of orchard grass at Terrebonne, (N)PKS fertilization did not increase forage yields compared with (N)PK fertilization (all main effects *P* > 0.16; **Table 3**; **Figure 3**). At Terrebonne, S fertilization increased forage yield (kg forage DM ha^-1^) in 2017 by 1,000 kg forage ha^-1^ (*P* = 0.0004) and in 2018 by 5,000 kg forage ha^-1^ (*P* < 0.0001); these forage yields were similar to PK- and PKS-fertilized alfalfa plots at Union. At Terrebonne, selenate amendment at 90 g ha^-1^ increased forage yields of NPK-fertilized grass plots in 2018, but not among NPKS-fertilized plots. There was a significant interaction between selenate amendment at 90 g ha^-1^ and S fertilization for orchard grass forage yield at Terrebonne in 2018 (*P* = 0.01).

(N)PKS fertilization without Se amendment decreased forage Se concentrations in high-producing forages (**Table 3** **;** **Figure 4**). Co-application of NPKS and selenate at 90 g ha^-1^ increased Se concentrations of grass-based forages in Union and in Terrebonne. At Terrebonne, co-application of NPKS and selenate increased harvested Se yields in 2017 (*P* = 0.03) and even more so in 2018 (*P* = 0.0007; **Figure 6**). In addition, co-application of NPKS and selenate increased Se agronomic efficiency (%) at Terrebonne in 2017 by 5 to 8% (*P* = 0.09) and in 2018 by 14 to 22% (*P* = 0.0005).

The effect of S fertilization on forage N concentrations differed by forage species and year (**Table 4**). Sulfur fertilization increased annual forage N concentrations for alfalfa at Union in 2017 (*P* = 0.05) and decreased it for orchard grass at Terrebonne in 2017 (*P* = 0.005) and 2018 (*P* < 0.0001). Despite decreasing forage DM N concentration, S fertilization increased forage N yield for orchard grass at Terrebonne in 2017 (*P* = 0.004) and 2018 (*P* < 0.0001) (**Figure 5**).

Orchard grass was the only forage species in which N agronomic efficiency was affected by S fertilization (all other *P* > 0.23). Sulfur fertilization of orchard grass at Terrebonne increased N agronomic efficiency (2017: *P*= 0.01; 2018: *P* < 0.0001), with the greatest responses observed for co-application of NPKS and 90 g Se ha^-1^).

The effect of S fertilization on forage S concentrations (forage DM %) and yields (kg S ha^-1^) differed by year and forage (**Table 5**). At Terrebonne, S fertilization increased forage S concentrations (2017: *P* = 0.0004; 2018: *P* < 0.0001) and yields (both *P* < 0.0001). At Union, S fertilization decreased forage S concentrations in 2017 (for grasses; *P* = 0.0005), but increased forage S concentrations in 2018 (for alfalfa: *P* = 0.005; for grasses: *P* = 0.07). Sulfur fertilization without Se amendment increased S yield of alfalfa at Union in 2018 (*P* interaction = 0.03; **Figure 6**). No changes were observed in Roseburg (all *P* > 0.13).

Sulfur agronomic efficiency was positively correlated with forage yield (r = +0.41; *P* = 0.0003) and negatively correlated with forage concentrations of N (r = -0.32; *P* = 0.006), S (r = -0.31; *P* = 0.007), and Se (r = -0.29; *P* = 0.01). Terrebonne was the only forage site consistently responsive to S fertilization (range of S agronomic efficiencies: 18-34%) and having a low variability (SED < 6%). In 2018, alfalfa was responsive to S fertilization without Se amendment (36%), but not with Se amendment.

## 4 Discussion

### 4.1 Forage requirements

Forages require light, water, heat, and nutrients for growth. We examined two forage grasses (i.e., orchard grass, and a grass mixture containing primarily tall fescue and orchard grass), one legume (i.e., alfalfa), and a grass-legume (50%:50%) mixture containing tall fescue, ryegrass, orchard grass and clovers). Forages were grown in varying climatic sites: Roseburg in southwestern Oregon, Terrebonne in central Oregon, and Union in eastern Oregon. In eastern, and even more so in central Oregon, the growing season is short and rain fall is limited and occurs mainly outside the growing season, requiring snow melt from the mountains to provide sufficient water in spring and irrigation during summer. Roseburg has a longer growing season, more precipitation, but requires irrigation in the summer. Cold, wet spring conditions can delay forage growth, which happened in May 2017.

Forages rely on plant availability of macro- and micro-nutrients in adequate amounts. The three most important macro-nutrients are N, P, and K (Tripathi et al., 2014). Nitrogen is a structural part of chlorophyll and plant proteins, most of which are in the leaves. Besides providing structure, plant proteins regulate water and nutrient assimilation, and thus are essential for plant growth (Masclaux-Daubresse et al., 2010). Grass-based forages are more susceptible to N deficiency than legume-based forages such as alfalfa, because forage legumes can convert N gas to ammonium-N compounds with the help of bacteria in the root region (Russelle et al., 1994; Moore et al., 2019). As expected, grass-based, unfertilized forages showed N deficiencies in both forage production years; the latter was indicated by low forage production, poor grass morphology (pale green color of leaves), and low forage grass DM N concentrations (<2-2.5% plant DM) (Moore et al., 2019). We conclude that grass-based forages across Oregon require N fertilization to prevent forage N deficiency. Furthermore, N fertilization is required to provide sufficient forage biomass for livestock.

Sulfur concentrations of unfertilized forages indicated that soil S availability was high in Roseburg, adequate in Union, and low in Terrebonne. Consequently, orchard grass at Terrebonne, but not the other two sites, required S fertilization. Roseburg’s soils are traditionally low in S because of their low sulfate-holding capacity; consequently, we were surprised about the high forage S concentrations at Roseburg. The S content of soil in Roseburg (western Oregon) is not traditionally measured because high rainfall makes it highly variable and single point measurements are not commonly used to advise the need for S fertilization. We did submit a soil sample from the Roseburg site to the same laboratory where S concentrations were determined in forage samples (Soil Health Laboratory at Oregon State University, Corvallis, OR). These results showed sulfate-S was 3.05 mg/kg. Oregon State University recommended soil sulfate-S concentrations are only given for alfalfa (15 mg/kg) and not grass or grass-clover forage crops. Based on a recommendation of 15 mg sulfate-S for grass and grass-legume forage crops, then Roseburg soils are S deficient. We also investigated the irrigation water for S content in Roseburg by reviewing data from the Oregon Department of Environmental Quality (AWQMS database) for average S concentrations upriver that was closest to the forage plots in Roseburg during summer irrigation season. The historical (1997 – 2022) average S concentrations was 6.68 mg sulfate/L, or 0.14 mEq/L. Using calculations for application of sulfate-S by irrigation water (Hopkins et al., 2007), this is equivalent to 14.5 kg ha^-1^ of S applied through the irrigation water. In Roseburg, the NPKS fertilization rate was 34 kg S ha^-1^. Thus, irrigation water resulted in approximately 50% of the recommended sulfate-S application. This explains why the high grass-clover S content in Roseburg indicated adequate S availability. At Union, unfertilized alfalfa had higher S concentrations than unfertilized grasses, which is consistent with the fact that alfalfa forages generally have higher macronutrient concentrations than grass forages (Saito, 2004).

Selenium concentrations of non Se-amended soil and forages were low across Oregon, which is consistent with findings in our previous studies (Oldfield, 2001; Hall et al., 2009; Hall et al., 2011a; Hall et al., 2013a; Brummer et al., 2014). We previously reported that Se concentrations of non Se-amended forages, similar to those observed in this report, results in low whole-blood Se concentrations and, consequently, poor health and growth of livestock consuming those forages (Hall et al., 2011b; Stewart et al., 2012; Hall et al., 2013a; Hall et al., 2013b; Hugejiletu et al., 2013; Hall et al., 2014a; Hall et al., 2014b; Hall et al., 2017; Hall et al., 2020). Forages assimilate soil Se species with different efficiencies (White, 2016). Selenate is highly mobile in the soil and is efficiently absorbed by the roots *via* high-affinity sulfate transporters (Pickering et al., 2000; White, 2018). Selenate is converted by soil microbes to SeMet and SeCys, which can be efficiently absorbed by the roots *via* amino acid membrane transporters (Pickering et al., 2000; White, 2018). Selenite is tightly bound to soil matter and cannot be easily absorbed by the roots (Pickering et al., 2000; White, 2018). Elemental Se or methylated Se cannot be absorbed by the roots (Pickering et al., 2000; White, 2018). Consequently, only a portion of soil Se species are available for Se assimilation by the plant. As a result, forage Se concentrations were lower than soil Se concentrations. We conclude that forage Se concentrations are a better indicator of plant available Se than soil Se concentrations.

### 4.2 Effects of selenate amendment on forage nutrient contents and Se amendment utilization

Springtime application of foliar sodium selenate amendment did not affect annual yields of forages (i.e., alfalfa, orchardgrass, or other grass mixtures) grown across Oregon. This agrees with previous studies performed by others when rates of up to 100 g of Se ha^-1^ were applied (reviewed by (Ramkissoon et al., 2019) showing that Se is not an essential element for plants (White, 2016). Applying Se as an amendment increases Se concentrations in the edible portions of crop plants. Selenium agronomic biofortification of plants has proven to be both effective and safe for alleviating Se deficiencies in human and livestock populations in many countries, e.g., Finland, New Zealand, Australia, United Kingdom, and others (Whelan et al., 1994; Makela et al., 1995; Broadley et al., 2006; Ramkissoon et al., 2019).

Despite being non-essential for plants, Se uptake may benefit plant growth and survival, e.g., by conferring tolerance to environmental factors associated with oxidative stress, and by providing resistance to pathogens and herbivory (Quinn et al., 2007; Pilon-Smits et al., 2009; White and Brown, 2010; El Mehdawi and Pilon-Smits, 2012; Feng et al., 2013; White, 2016). In our study, selenate amendment at 45 g ha^-1^ increased forage biomass of non-fertilized grasses at Union and Terrebonne. We conclude that Se-amendments may benefit growth of grass pastures that are low in plant available macro-nutrients.

We showed in our previous report (Wang et al., 2021) that forage Se concentrations increased linearly from 2.06 to 4.15 mg kg^-1^ forage dry matter (DM) with doubling of foliar sodium selenate application rates from 45 to 90 g Se ha^-1^. We concluded that selenate amendment rates can be used to predict forage Se concentrations. In turn, feeding selenate amended forages to livestock can prevent Se-deficiency.

Of the absorbed selenate, up to 82% was converted to SeMet in the forage, indicating efficient conversion of selenate to SeMet in plants (Wang et al., 2021). The conversion of selenate to SeMet depended on forage growth and time span between Se amendment application and forage harvest. Almost all the incorporated Se was removed during the growing season with forage harvesting (87% and 9% in the first and second cuts, respectively), indicating a limited selenate holding capacity of the soils. Grass-based forages had greater increases in Se concentrations in the first two cuts after selenate amendment compared with legume-based forages.

In this report, we calculated Se agronomic efficiencies after selenate amendment (indicative of Se utilization by the plant). The variability of Se agronomic efficiencies was low, indicating that the foliar spraying application was uniform. We conclude that application of foliar selenate amendment is consistent in improving forage Se concentrations.

We also showed that Se uptake increased with forage growth. The four forages (i.e., alfalfa, grass mixtures, orchard grass, and grass-clover) are non Se-accumulating forages (cannot tolerate tissue Se concentration >10 to 100 µg Se g^-1^ plant DM) (White, 2016). Non Se-accumulating forages have three Se pools in the plant: vacuole-stored selenate, proteins containing selenoamino acids (SeAA), and non-proteinogenic organo-selenium compounds (e.g., methylated selenocysteine). Plants usually absorb inorganic selenate either through the roots or through the leaf stomata if foliar applied. The inorganic selenate pool serves as a reserve pool for SeAA synthesis, which is promoted by plant protein synthesis (Pickering et al., 2000; White, 2018). Our previous study indicated that the inorganic selenate pool is limited to approximately 5 mg Se kg^-1^ forage DM, and cannot be increased, for example by a 10× higher selenate amendment rate than used in the current study (Hall et al., 2023). In this report, Se utilization by the plant was not altered by selenate amendment rate, indicating an efficient absorption of selenate by the plant. Furthermore, selenate was efficiently converted in the plant to SeMet (Wang et al., 2021). This conversion requires N-containing enzymes. We conclude that Se incorporation into forage plants depends on efficient conversion of N and Se into plant proteins, which, in turn, depends on weather conditions, macronutrient availability, and forage species.

Selenate amendment did not impact concentrations and yields of N and S in non-fertilized forages. This was not surprising given that much lower amounts of selenate were applied compared with plant available amounts of S and N in the soil. Moreover, selenate was foliar amended rather than soil amended, facilitating leaf absorption of selenate.

### 4.3 Effects of NPK or PK fertilization on forage nutrient contents and fertilizer utilization

Fertilization with NPK or PK (for alfalfa) increased biomass yields of forages grown across Oregon in both years. Forages require N for synthesis of nucleotides, ATP, proteins, and chlorophyll (Masclaux-Daubresse et al., 2010). The largest biomass increases were observed for orchard grass at Terrebonne, the forage site with the lowest soil nitrate concentrations in 2017. Forage grasses utilize more N during the early vegetative stages for tillering than legumes do for early shoot growth (Masclaux-Daubresse et al., 2010; Moore et al., 2019). Orchard grass received the most N fertilizer (403 kg N ha^-1^ *vs*. <145 kg N ha^-1^ at the other sites). We conclude that NPK or PK (for alfalfa) fertilization increases forage productivity in Oregon.

Nitrogen fertilization rates were adapted to meet local forage and soil nitrogen requirements. Nitrogen is primarily applied in the form of ammonium and nitrate compounds. We used granular ammonium phosphate in spring and urea or slow-release urea after each forage cut for N fertilizer. Ammonium ion and ammonia, the urease hydrolysis products of urea, are primarily absorbed by the roots through ammonium- and ammonia-specific transmembrane proteins (Masclaux-Daubresse et al., 2010). Ammonium can only be stored in limited amounts in vacuoles before it is toxic to plants. Consequently, ammonium ion is rapidly transported to the leaves, where it is incorporated into DNA, ATP, and amino acids. N fertilizer rates met soil N requirements, shown best by soil nitrate concentrations in 2018. Based on forage N concentrations and plant morphology, N fertilization rates also met plant nitrogen requirements. Nitrogen fertilizer increased the (inadequate) forage N concentrations of non-fertilized forage grasses at Union (144 kg N ha^-1^ total application rate) and at Terrebonne (403 kg N ha^-1^ total application rate), but not the (adequate) forage N concentrations of non-fertilized forage legumes for grass-clover at Roseburg (140 kg N ha^-1^ total application rate) and alfalfa at Union (0 kg N ha^-1^ total application rate). Nonetheless, N fertilizer amounts were not enough to raise N concentrations of fertilized grasses to levels that would meet crude protein needs (15 to 18%) for livestock consuming the forages at Union and at Terrebonne. Our data support previous studies (Hart et al., 2000; Pirelli et al., 2004; Moore et al., 2019) showing that regular testing of forage N concentrations can aid in determining appropriate N fertilization rates for increasing forage production and N concentrations.

In this study, we calculated N agronomic efficiency after fertilization as an indication of N uptake and utilization. We showed that N fertilization consistently increased forage N uptake, and resulted in increased harvested forage N amounts after fertilizer treatment. Based on the positive correlation between forage yield and N agronomic efficiency, fresh forage growth promotes N fertilizer uptake, and more ammonium fertilizer is incorporated into forage proteins. Fresh forage growth depends on the photosynthetic capacity of forages, which, in turn, depends on light, water, sufficient leaf cover, and warm soil temperatures. We conclude that weather conditions have to be considered when determining N fertilization rates for forage production.

Fertilization with NPK or PK increased uptake and utilization of S in forages and increased harvested amounts of forage S. Sulfur is primarily absorbed by sulfate-specific transmembrane proteins in the root in the form of sulfate and then transported to the leaves (Saito, 2004). In the leaves, sulfate is converted into S-containing amino acids (i.e., Cys and Met) for protein synthesis. The correlation results indicate that forage growth promotes sulfate uptake and synthesis of Cys and Met to meet the plant S requirements. Fertilization with NPK increased sulfate uptake in grasses even more so in combination with 90 g ha^-1^ Se amendment, suggesting that the higher application rate of foliar selenate amendment may promote transmembrane protein synthesis in the roots for sulfate absorption when there is inadequate S available to the plant. Fertilization with NPK or PK fertilizers increased forage S concentrations in S-sufficient plant environments (i.e., Roseburg and Union) and lowered forage S concentrations in S-deficient plant environments (i.e., Terrebonne), indicating that NPK or PK fertilization only increased forage S concentrations when sufficient sulfate was available to roots.

Fertilization with NPK increased harvested amounts of forage Se by increasing selenate uptake and metabolism. Foliar selenate amendment is taken up by the leaves and converted into Se-containing amino acids (i.e., SeCys and SeMet) for protein synthesis (White, 2016). Correlation results indicate that forage growth promotes foliar selenate uptake and utilization by the plants. Co-application of NPK and 90 g ha^-1^ Se amendment resulted in the largest increases in forage Se yields. At Terrebonne, NPK-fertilization and each additional 45 g ha^-1^ Se amendment increased Se yield by 5 g Se ha^-1^ plant DM to a total of 20 g Se/kg plant DM in NPK-fertilized and 90 g ha^-1^ Se amended plots. We hypothesize, and our data supports, that in the absence of sufficient plant available S, Se can act as a S substitute making selenocysteine instead of cysteine and selenomethionine instead of methionine for protein synthesis.

Forage Se concentrations depended on both plant response to (N)PK fertilization and plant available Se concentrations. Fertilization with NPK did not affect forage Se concentrations in the absence of Se amendment, indicating that NPK fertilization promoted Se uptake by the roots. Fertilization with NPK or PK fertilizers did not affect forage Se concentrations in selenate-amended S-sufficient forage sites (i.e., Roseburg and Union), suggesting that NPK or PK fertilizers promoted foliar selenate amendment uptake. In contrast, NPK-fertilization plus selenate amendment lowered forage Se concentrations in S-deficient forage sites (i.e., Terrebonne). It is possible that higher selenate amendment rates are needed to increase forage Se concentrations in S deficient sites.

### 4.4 Effects of NPKS or PKS fertilization on forage nutrient utilization

Co-application of S with NPK or PK (for alfalfa) only increased biomass yields of orchard grass at Terrebonne in central Oregon, which had the lowest plant S concentrations at baseline. Sulfur is considered the fourth most important macro-nutrient (behind NPK) with many essential functions (Tripathi et al., 2014). For example, S-containing peptides and proteins are important for chlorophyll synthesis and function; for legume root nodule formation needed for N gas assimilation; for synthesis of amino acids, enzymes, and vitamins; for plant detoxification processes; for redox chemistry; and for disease resistance (Saito, 2004; Zenda et al., 2021).

Sulfur is becoming more important as a limiting nutrient in forage production, as S dioxide concentrations in the air have decreased over the last several decades by over 90%. Terrebonne and Roseburg are more susceptible to plant S deficiency because of their coarse soil texture (>50% sand content) and low organic matter content (<2%), which makes soils susceptible to sulfate leaching. Thus, we applied more S in Terrebonne and Roseburg (34 kg S ha^-1^) than in Union (12 kg S ha^-1^). The resulting high grass-clover S content in Roseburg indicated adequate S availability, from irrigation water as well as S fertilizer.

The only biomass yield increases observed for NPKS-fertilized orchard grass were at Terrebonne, the forage site that received the most N fertilizer (403 kg N ha^-1^ *vs*. <145 kg N ha^-1^ at the other sites) and the site that had the lowest soil nitrate concentrations in 2017. Forage requirement for S and N are closely associated, as both are required for protein and chlorophyll formation and function, as well as for absorption and assimilation of nutrients (Saito, 2004; Tripathi et al., 2014; Zenda et al., 2021). We used ammonium sulfate and gypsum (hydrated calcium sulfate) for S application. Sulfur application rates were based on forage production goals; soil and forage analyses for S; soil texture and organic matter content; NPK fertilization rates; and weather conditions. We conclude that NPKS fertilization is necessary for maximizing forage production in Se depleted soils with limited plant sulfate availability, but with large forage growth potential.

Co-application of sulfate and NPK only increased forage sulfate concentrations when weather conditions were optimal for forage growth, and/or when plant sulfate availability was limited by the soil, by high forage growth rate, or by both. At Roseburg, despite limited soil sulfate availability because of sandy soil texture, low organic matter content and low CEC capacity, sulfate fertilization did not affect the already high forage S concentrations. Sulfur fertilizer uptake was below 8% with no changes in S yield, indicating no need for sulfate fertilization beyond what was applied *via* irrigation water. At Terrebonne, sulfate fertilization increased orchard grass S concentrations and yield. Sulfur fertilizer uptake was at least 18% and was higher in 2018 than in 2017. However, S concentrations of NPKS-fertilized orchard grass remained under 2%, which indicated that higher sulfate application rates than were applied are needed in high forage production years. At Union, where soil had higher organic matter and less sand, sulfate application increased alfalfa and grasses sulfate concentrations in 2018 but not in 2017, indicating that sulfate application may be needed in high forage production years, but not in low forage production years such as in 2017.

The co-dependency of S and N for forage uptake was exemplified by the effect of S application on forage N concentrations and N uptake as well as the high correlation between the forage S and N concentrations. At Terrebonne, sulfate fertilization increased N fertilizer uptake by orchard grass. The decrease in orchard grass N concentrations indicated that higher NPK fertilizer rates were needed to optimize forage growth and quality in high forage production years such as in 2018. We conclude that S fertilization increases N requirements in low plant available S soils. At Roseburg, sulfate application did not affect forage N concentrations and N uptake, indicating that sulfate application was not needed in either forage production year, as sufficient sulfate was available *via* irrigation water. At Union, S application had minimal effects on alfalfa N concentrations and N uptake, as sufficient sulfate was available in the soil.

Of great concern is the competition between sulfate and selenate for root uptake in forages (Moore et al., 2019). Selenate is highly mobile in soil and highly available to plants, being taken up *via* the same high-affinity sulfate-S membrane transporters (Li et al., 2008; Luo et al., 2019). In our study, S application decreased forage Se concentrations, when plant availability of Se was low. Similarly, Stroud et al. (Stroud et al., 2010) showed in wheat that S fertilization decreased Se-uptake in the absence of Se-application. One potential reason for this is that S supplementation alone may decrease the expression of genes encoding plant S transporters (Stroud et al., 2010; Boldrin et al., 2016; White, 2018). Another potential reason is that sulfate fertilizer out-competes soil selenate for root uptake. We conclude that sulfate fertilization decreases forage Se concentration of non-selenate amended fields.

In our study, the effect of co-application of foliar selenate with NPKS on forage selenate uptake depended on forage available S and/or forage species. At Terrebonne, S fertilization increased selenate amendment uptake in both years. Similarly, other researchers have shown that co-application of foliar Se with NPKS fertilizers doubled the Se concentration in wheat grains compared with application of foliar Se alone (Ramkissoon et al., 2019). One potential reason is that increased forage growth in response to S fertilization may promote foliar selenate uptake and/or utilization. Sulfur fertilization did not affect selenate amendment uptake or forage Se concentrations at the other two forage sites where S fertilization was not required. Of concern were the lower forage Se concentrations, lower annual Se yields, and lower Se agronomic efficiencies after selenate amendment at Roseburg in 2018, which was associated with higher forage S concentrations and S yields even without S fertilization at that site. We conclude that excess sulfate application *via* irrigation water may be detrimental to forage Se concentrations in selenate amended plots.

## 5 Conclusion

Application of foliar selenate amendment increases forage Se concentration based on amendment rates, irrespective of forage fertilization practices. Plant uptake of N and S from fertilizers did not interfere with plant uptake of selenate amendment. In fact, foliar selenate amendment synergizes with NPK(S) fertilization in promoting forage biomass production and plant uptake of N and S from fertilizer to satisfy nutrient requirements. However, S fertilizers can decrease forage Se concentrations, when plant available Se is already low and no selenate is amended. We have shown that multiple factors affect forage Se concentrations and Se yields including selenate amendment rate, the amount of forage biomass produced, forage species of interest, soil characteristics, and changing weather conditions from year to year. Because selenate amendment and S application cost extra, and S has the capacity to acidify soils, their concentrations in soil and plants should be routinely measured before application. Combining springtime sodium selenate foliar application with NPKS/PKS fertilizers at amounts adapted to meet local forage and soil requirements is an effective strategy to maintain optimal forage growth and quality on low Se soils in Oregon.

## Data availability statement

The original contributions presented in the study are included in the article/supplementary material. Further inquiries can be directed to the corresponding author.

## Author contributions

JH: Conceptualization, Project Administration, Investigation, Formal analysis, Writing - Original Draft. GB: Data Curation, Formal analysis, Writing - Original Draft, Visualization. SF: Investigation, Writing - Review and Editing. GP: Conceptualization, Funding acquisition, Project Administration, Writing - Review and Editing. MB: Investigation, Funding acquisition, Supervision, Writing - Review and Editing. GW: Investigation, Writing - Review and Editing. TD: Investigation, Resources, Writing - Review and Editing. GB: Investigation, Resources, Validation, Writing - Review and Editing. All authors contributed to the article and approved the submitted version.
